# Impact of a *NDUFC2* Variant on the Occurrence of Acute Coronary Syndromes

**DOI:** 10.3389/fcvm.2022.921244

**Published:** 2022-05-31

**Authors:** Giovanna Gallo, Serena Migliarino, Maria Cotugno, Rosita Stanzione, Simone Burocchi, Franca Bianchi, Simona Marchitti, Camillo Autore, Massimo Volpe, Speranza Rubattu

**Affiliations:** ^1^Cardiology Unit, Department of Clinical and Molecular Medicine, School of Medicine and Psychology, Sant'Andrea Hospital, Sapienza University of Rome, Rome, Italy; ^2^Division of Cardiology, Department of Medical and Surgical Science, Magna Graecia University, Catanzaro, Italy; ^3^IRCCS Neuromed, Pozzilli (IS), Italy; ^4^Cardiology Unit, Belcolle Hospital, ASL Viterbo, Viterbo, Italy

**Keywords:** acute coronary syndrome, genetic, *NDUFC2*, complex I, mitochondrial dysfunction

## Abstract

**Background:**

Among several potential mechanisms, mitochondrial dysfunction has been proposed to be involved in the pathogenesis of coronary artery disease (CAD). A mitochondrial complex I deficiency severely impairs cardiovascular health and contributes to CAD development. Previous evidence highlighted a key role of *NDUFC2*, a subunit of complex I, deficiency in the increased occurrence of renal and cerebrovascular damage in an animal model of hypertension, and of juvenile ischemic stroke occurrence in humans. Furthermore, a significant decrease of *NDUFC2* mRNA was detected in peripheral blood mononuclear cells from patients experiencing acute coronary syndrome (ACS). The T allele at *NDUFC2*/rs23117379 variant is known to associate with reduced gene expression and mitochondrial dysfunction.

**Objective:**

In the present study we tested the impact of the T/C *NDUFC2*/rs23117379 variant on occurrence of ACS in a prospective cohort of CAD patients (*n* = 260).

**Results:**

Hypertension, smoking habit, diabetes and hypercholesterolemia were present in a large proportion of patients. Non-ST-elevation myocardial infarction (NSTEMI) represented the most frequent type of ACS (44%, *n* = 115), followed by ST-elevation myocardial infarction (STEMI) (34%, *n* = 88) and unstable angina (22%, *n* = 57). The alleles/genotypes distribution for T/C at *NDUFC2*/rs23117379 revealed that the TT genotype was associated with a trend toward the development of ACS at an earlier age (TT 61 ± 12, CT 65 ± 12 and CC 66 ± 11 years; *p* = 0.051 after adjustment for gender, hypertension, smoking habit, diabetes and hypercholesterolemia) and with a significant predictive role for ACS recurrence (hazard ratio [HR]1.671; 95% confidence interval [CI], 1.138–2.472; *p* = 0.009).

**Conclusions:**

Our findings are consistent with a deleterious effect of *NDUFC2* deficiency on acute coronary events predisposition and further support a role of the *NDUFC2*/rs23117379 variant as a genetic cardiovascular risk factor.

## Introduction

Coronary artery disease (CAD) represents the most common cause of cardiovascular (CV) death worldwide. Prevention and treatment of traditional CV risk factors, such as hypertension, dyslipidemia, diabetes, and smoking, represent the most relevant strategy to fight the occurrence of CVD (1). Genetic factors are also known to play a contributory role (2).

Over the last few years, several efforts have been made to identify molecular mechanisms involved in the development and progression of atherosclerotic plaques and in triggering acute inflammatory processes potentially contributing to plaque instability. Among others, a role of mitochondrial dysfunction in the development of atherosclerosis has been shown (3–6). Of note, mitochondrial complex I deficiency was detected in patients with acute coronary syndromes (ACS), with a consequent significant increase of reactive oxygen species (ROS) levels, reduced adenosine triphosphate (ATP) levels and a higher degree of mitochondrial structural damage and dysfunction (7–9).

In this context, the *NDUFC2* (NADH dehydrogenase [ubiquinone] 1 subunit) has emerged as a key fundamental subunit of mitochondrial complex I that is needed for the appropriate assembly and activity of the complex. *In-vitro, NDUFC2* disruption in vascular cells alters mitochondrial complex I assembly and activity, reducing mitochondrial membrane potential and ATP levels and increasing ROS production and inflammation (7, 10). *In-vivo, Ndufc2* silencing may contribute to cause increased cerebral and renal vascular damage in an animal model of hypertension (11). In humans, *NDUFC2* messenger ribonucleic acid (mRNA) level was significantly down-regulated, along with the expression of antioxidant molecules such as uncoupling protein 2 (UCP2) and superoxide dismutases 1 and 2 (SOD1, SOD2), at the time of ACS occurrence (7). Moreover, the T allele at *NDUFC2*/rs11237379 variant, which is associated with a significant reduction of gene expression (10), is accompanied by increased occurrence of early-onset ischemic stroke through a recessive mode of transmission (11). The *NDUFC2*/rs11237379 variant is commonly represented in the general population (12).

The present study was performed to evaluate (1) the impact of the carrier status of the T vs. C allele at *NDUFC2*/rs11237379 variant on the occurrence of a first ACS episode, and (2) the long-term prognostic impact of T vs C allele on the incidence of recurrent ACS episodes in a prospective cohort of Caucasian CAD patients.

## Methods

This was a single-center prospective study that included patients affected by CAD admitted and followed at the Unit of Cardiology, S.Andrea Hospital in Rome, Italy, between April 2009 and May 2016.

The inclusion criteria were ACS episodes [unstable angina, non-ST-segment elevation MI (NSTEMI), ST-segment elevation MI (STEMI)] as first manifestation of CAD, diagnosed based on standard criteria (13, 14); evidence of critical coronary artery stenosis in at least one vessel (70%), as documented by coronary angiography; availability of a well-documented clinical follow-up after the first ACS episode. Exclusion criteria were lack of coronary angiography, of a well-documented clinical follow-up, of availability of most of the parameters selected for statistical analysis; patients with neoplasia and short life expectancy (<6 months).

**Figure 1:**
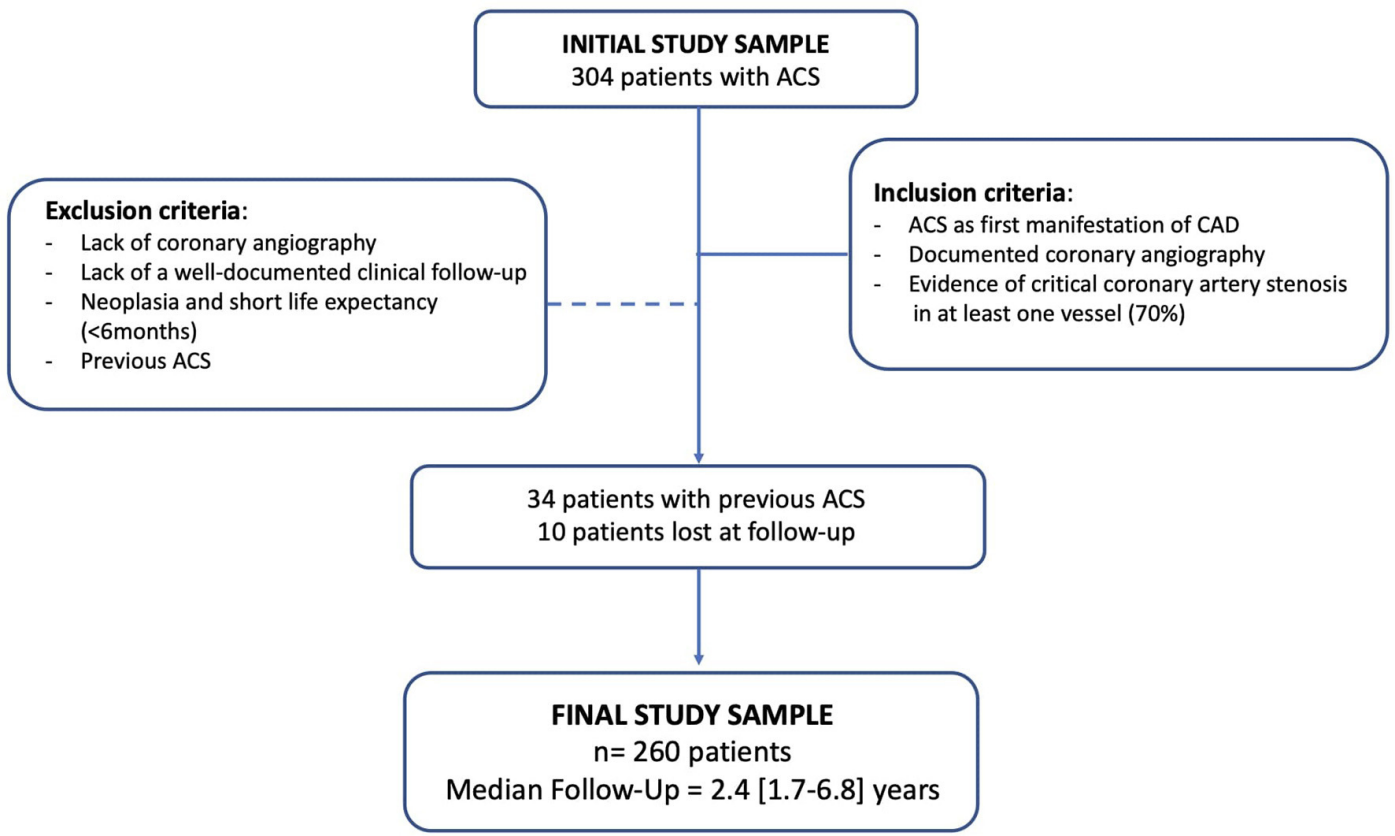
Flow chart of the study. ACS, acute coronary syndrome; CAD, coronary artery disease.

Out of 304 originally enrolled patients, 260 individuals had their hospitalization for a first ACS episode at our institution and were subsequently followed. The remaining 44 patients had already experienced a first ACS episode before admission at our hospital (*n* = 34) or were lost at follow-up (*n* = 10). A total of 260 CAD patients were finally included in the analysis (Figure 1).

The following parameters were recorded for each patient: demographic data, presence/absence of hypertension, hypercholesterolemia, diabetes, tobacco use, prescribed medications, data on cardiac geometry and function, type of coronary revascularization following the first ACS episode.

Hypertension was diagnosed based on the World Health Organization/International Society of Hypertension criteria and if subjects were routinely receiving antihypertensive therapy (15, 16). Hypercholesterolemia was defined by a total cholesterol blood level higher than 220 mg/dl or routinely use of lipid lowering drugs. Type 1 and 2 diabetes mellitus were diagnosed according to the American Diabetes Association (ADA) Guidelines and/or if subjects were receiving antidiabetic therapy (17, 18). Smoking habit was also recorded (i.e., smokers were considered former only if they had stopped smoking >2 months before entering the study). Left ventricle (LV) internal diameters, wall thickness, LV systolic and diastolic function were measured according to the guidelines of the American Society of Echocardiography and European Association of Cardiovascular Imaging (19).

At the time of recruitment, each patient, after providing informed written consent, underwent a venous blood sample drawing for assessment of the carrier status of the T/C allele at *NDUFC2*/rs11237379. The study was approved by the Ethical Committee of the S. Andrea Hospital (1916_09).

Following the first ACS episode, clinical follow-up of each patient was well documented. Patients performed periodic check-up visits with a frequency of 1–2 per year. Follow-up data of all patients were obtained through review of ambulatory visits, phone calls and careful revision of medical records. Thus, a detailed and complete cardiac history, with available information regarding re-hospitalizations, new revascularization procedures and medical treatments administered over the years, was available for all patients included in the study.

Genomic DNA was extracted by a commercially available kit (Qiagen, Milan, Italy). The *NDUFC2*/rs11237379 variant was characterized by a previously reported procedure (11). In particular, the real-time polymerase chain reaction (RT-PCR) was performed by a TaqMan technology assay (Life Technologies) using the ViiA 7 Real-Time PCR System (Applied Biosystem, Foster City, CA, USA).

### Statistical Analysis

Data analysis regarding sample characteristics was performed with SPSS software package (version 25.0, SPSS Inc, Chicago, Illinois, USA). Continuous variables are expressed as mean standard deviation; categorical variables are expressed with the corresponding frequencies and percentages.

Differences between groups were analyzed either by *t*-test or 1-way analysis of variance (ANOVA) if groups were more than two. The Bonferroni *post-hoc* least significant difference test was performed to complete the analysis for multiple comparisons. To evaluate whether the *NDUFC*2/rs11237379 variant was independently associated with a younger age at first ACS presentation, a covaried 1-way ANOVA was performed considering age, gender, hypertension, diabetes mellitus, dyslipidemia, and smoking habit as covariates and considering the variant as an independent value. Chi-square test was used for categorical variables. Genotype frequencies were evaluated and Hardy–Weinberg equilibrium (HWE) was tested using Pearson's Chi-square test. The assumption of both dominant (score of 0 for CC genotype, 1 for CT and TT genotypes) and recessive (score of 0 for combined CC and CT genotypes, 1 for TT genotype) models for genotype analysis was considered.

At univariate analysis by Cox regression model, the following predictors were considered: *NDUFC*2/rs11237379 TT genotype carrier status, sex, age at first event, diabetes, hypercholesterolemia, hypertension, treatment with beta-blocker, acetylsalicylic acid, calcium channel blocker, angiotensin-converting enzyme inhibitor (ACEI), AT1 receptor blocker (ARB), other antiplatelet drugs, statin.

Multivariate models were selected using a forward stepwise regression method based on optimization of the Akaike Information Criterion, finally selecting the following covariates: *NDUFC*2/rs11237379 TT genotype status, diabetes, treatment with statin.

A value of P < 0.05 was chosen as the cut-off level to declare statistical significance in all analyses.

## Results

Table 1 shows the main clinical characteristics of the study cohort and the risk factors distribution at the time of the first ACS episode. Male patients represented 66% of the cohort (172 subjects) with a mean age of 64 ± 12 years. A large percentage of patients were hypertensive (71.9%, *n* = 187), either smokers or ex-smokers (66%, *n* = 172), affected by diabetes (26%, *n* = 68) and by hypercholesterolemia (53.8%, *n* = 140). NSTEMI represented the most frequent type of ACS (44%, *n* = 115), followed by STEMI (34%, *n* = 88) and unstable angina (22%, *n* = 57).

**Table 1:** General characteristics of the study sample (*n* = 260 patients).

**General data**	
Male (%)	172 (66)
Age (years)	64 ± 12
Smoking habit (%)	68 (26)
Ex-smokers (%)	88 (33.8)
Hypertension (%)	188 (72)
Dyslipidemia (%)	140 (53.8)
Diabetes (%)	68 (26)
Aspirin (%)	115 (44)
Beta blockers (%)	176 (67.7)
Statins (%)	125 (48)
ACE inhibitors/ARBs (%)	150 (57.6)
Calcium channels blockers (%)	44 (17)
Diuretics (%)	76 (29.2)
Insulin (%)	25 (9.6)
Oral glucose lowering agents (%)	49 (18.8)
LVEF %	48 ± 12
Total cholesterol (mg/dL)	190 ± 45
LDL-c (mg/dL)	138 ± 24
HDL-c (mg/dL)	35 ± 13
Triglycerides (mg/dL)	137 ± 38
Glycaemia (mg/dL)	105 ± 20
Creatinine (mg/dL)	1.05 ± 0.3
eGFR (mL/min/1.73 m^2^)	65 ± 11
STEMI (%)	88 (34)
NSTEMI (%)	115 (44)
Unstable angina (%)	57 (22)
Percutaneous coronary intervention (%)	169 (65)
Coronary artery bypass graft (%)	91 (35)

Regarding the T/C alleles distribution at *NDUFC*2/rs11237379 variant, 45.4% of the enrolled subjects were carrier of the C allele and 54.6% carried the T allele. The frequency of the observed genotypes was 22.3% for CC genotype (*n* = 58), 46.5% for CT genotype (*n* = 121) and 31.2% for TT genotype (*n* = 81); chi-square test: *p* > 0.05 respecting the HWE.

There were no significant differences among genotype groups regarding gender (*p* = 0.064), prevalence of hypertension (*p* = 0.908), smoking habit (*p* = 0.075), diabetes (*p* = 0.268) and hypercholesterolemia (*p* = 0.145) (Table 2). In addition, no significant difference was detected between the groups regarding the number of diseased coronary arteries (*p* = 0.645), the type of performed coronary revascularization (*p* = 0.274) and the type of implanted coronary stents (*p* = 0.185) (Table 2).

**Table 2:** Main clinical variables of the study sample according to *NDUFC2/*rs11237379 genotype at the time of a first ACS occurrence.

**Variables**	**CC**	**CT**	**TT**	***p*-value**
	***n* = 58**	***n* = 121**	***n* = 81**	
Male (%)	39 (67)	80 (66)	53 (69)	0.064
Age (years)	66 ± 11	65 ± 12	61 ± 12	0.018
Smokers (%)	16 (28)	29 (24)	23 (34)	0.075
Hypertension (%)	42 (73)	89 (73)	57 (73)	0.908
Dyslipidaemia (%)	30 (52)	71 (58)	39 (51)	0.145
Diabetes (%)	15 (26)	37 (31)	16 (21)	0.268
Aspirin (%)	27 (46)	53 (44)	35 (45)	0.524
Beta blockers (%)	40 (67)	82 (66)	54 (68)	0.263
Statins (%)	28 (47)	59 (48)	38 (48)	0.612
ACE inhibitors/ARBs (%)	34 (57)	70 (57)	46 (58)	0.187
Calcium channels blockers (%)	10 (17)	24 (20)	10 (13)	0.432
Diuretics (%)	18 (29)	34 (27)	24 (28)	0.321
Insulin (%)	6 (10)	11 (9)	8 (8)	0.785
Oral glucose lowering agents (%)	11 (19)	22 (17)	16 (21)	0.643
LVEF, %	49 ± 12	47 ± 11	48 ± 11	0.378
Total cholesterol (mg/dL)	194 ± 41	197 ± 43	202 ± 46	0.245
LDL-c (mg/dL)	144 ± 26	137 ± 22	140 ± 24	0.169
HDL-c (mg/dL)	37 ± 13	39± 12	34 ± 11	0.758
Triglycerides (mg/dL)	138 ± 39	144 ± 35	141 ± 36	0.362
Glycaemia (mg/dL)	104± 21	108 ± 20	105± 18	0.823
Creatinine (mg/dL)	1.05± 0.4	1.06± 0.2	1.04± 0.3	0.295
eGFR (mL/min/1.73 m^2^)	65± 11	64± 12	67± 11	0.09
STEMI (%)	20 (35)	39 (32)	29 (35)	0.08
NSTEMI (%)	24 (42)	59 (49)	32 (40)	0.194
Unstable angina (%)	14 (23)	23 (19)	20 (25)	0.065
Number of diseased coronary arteries (%)				
1 vessel CAD	24 (41)	54 (45)	34 (42)	0.645
2 vessel CAD	20 (35)	37 (31)	27 (33)	
3 vessel CAD	14 (24)	30 (24)	20 (25)	
Percutaneous coronary intervention (%)	39 (68)	81 (66)	49 (65)	0.274
Coronary artery bypass graft (%)	19 (32)	40 (34)	27 (35)	
Type of coronary stents (%)				
Bare metal stents (%)	17 (44)	34 (42)	20 (41)	0.185
Drug eluting stents (%)	22 (56)	47 (58)	29 (59)	

The carrier status of the TT genotype was associated with a younger age at the first ACS event compared to subjects carrying the CT and CC genotypes (61 ± 12, 65 ± 12 and 66 ± 11 years, respectively; *p* = 0.018). After adjustment for gender, hypertension, smoking habit, diabetes and hypercholesterolemia, we observed a trend toward statistical significance (*p* = 0.051).

The median follow-up time was 2.4 years (interquartile range 1.7–6.8 years). During the follow-up period, recurrent ACS episodes were experienced by 25 patients in the CC genotype group (43.1%), 53 subjects in the CT genotype group (43.8%) and 47 in the TT genotype group (58%). Among the 125 patients who experienced recurrent ACS, 75 were treated with PCI (60%) and 50 with CABG (40%), without significant differences among groups (*p* = 0.655).

Presence of the T allele associated with increased occurrence of recurrent ACS by a recessive mode of transmission (odds ratio [OR] 2.7, 95% confidence interval [CI] 1.39–5.26; *p* = 0.003).

At univariate analysis, the TT genotype carrier status and diabetes showed a significant impact on recurrent ACS (Table 3), maintaining a significant predictive role also at multivariate Cox analysis (Table 4). The treatment with statins was confirmed as a significant protective factor toward ACS recurrence over the follow-up time (Tables 3, 4).

**Table 3:** Predictors of ACS recurrence at follow-up by univariate analysis.

	**H.R. (95% C.I.)**	***P* values**
*NDUFC*2/rs11237379 TT genotype	1.480 (1.028–2.137)	0.035
Diabetes	1.519 (1.030–2.239)	0.039
Hypertension	1.517 (0.860–2.677)	0.150
Dyslipidaemia	1.268 (0.592–1.652)	0.387
Smoking habit	0.943 (0.745–1.193)	0.625
Sex	0.967 (0.598–1.563)	0.210
Age	1.009 (0.993–1.026)	0.255
LVEF	0.640 (0.277–1.180)	0.781
Aspirin	1.179 (0.430–3.233)	0.749
Beta-blockers	1.054 (0.611–1.818)	0.851
ACEi/ARBs	0.981 (0.762–1.165)	0.456
Calcium channel blockers	1.267 (0.735–2.183)	0.394
P2Y12 inhibitors	0.983 (0.906–1.067)	0.686
Statins	0.590 (0.390–0.894)	0.013
Oral glucose lowering agents	1.241 (0.636–2.423)	0.526
Insulin	0.897 (0.460–1.748)	0.749

**Table 4:** Predictors of ACS recurrence at follow-up by multivariate analysis.

	**H.R. (95% C.I.)**	***P* values**
*NDUFC*2/rs11237379 TT genotype	1.671(1.138–2.472)	0.009
Diabetes	1.494 (1.011–2.208)	0.044
Statins	0.574 (0.347–0.598)	0.031

## Discussion

The major goals of the present study were to investigate (1) the impact of the carrier status of the T vs. C allele at *NDUFC2*/rs11237379 variant on the occurrence of a first ACS episode and (2) the prognostic impact of T vs. C allele on the occurrence of new coronary events (unstable angina, NSTEMI, STEMI) in CAD patients. The results of the study demonstrate that ACS tended to occur at a younger age in patients carrying the TT genotype and, most importantly, that carrier status for the TT genotype was a significantly independent risk factor for ACS recurrence in an Italian cohort of CAD patients. The frequency of the TT genotype was 29% in our cohort, consistent with previous reports (11, 12). Notably, the T allele at this variant is known to associate with reduced *NDUFC2* expression (10, 11).

As previously shown, the downregulation of *NDUFC2* is responsible of an impairment of the complex I assembly leading to a reduction of its activity, decreased ATP synthesis and increased ROS generation (10, 11). In turn, the accumulation of ROS is known to promote mitochondrial protein and DNA damage with subsequent alterations of mitochondrial structure and function and further increase of ROS levels. At the vascular level, the accumulation of ROS causes a reduction of nitric oxide bioavailability, which compromises vascular relaxation and favors the atherosclerosis development (20, 21). The latter underlies the decrease of coronary and cerebral blood flow ultimately producing organ and tissue ischemia. More specifically, *NDUFC2* downregulation in vascular cells is reported to cause deleterious effects on cell viability, impairing angiogenesis, and stimulating the release of molecules involved in atherogenesis and plaque instability (10, 22).

*NDUFC2* downregulation was first discovered as a contributory mechanism to cerebral and renal vascular damage upon a high-salt Japanese-style diet (JD) in the animal model of stroke-prone spontaneously hypertensive rat (SHRSP) (11). The same renal and cerebrovascular injuries developed in the animal model of JD-fed spontaneously hypertensive stroke-resistant rat (SHR-SR) once subjected to a heterozygous deletion of *Ndufc2* (11).

In humans, the T allele variant at *NDUFC2*/rs11237379 was associated with increased occurrence of early-onset ischemic stroke by a recessive mode of transmission (11). The functional significance of rs11237379 was documented by its direct relationship with gene expression level, with the T allele being significantly associated with reduced gene expression (11). Consistently, another study documented that the reduced gene expression associated to the TT genotype led to increased oxidative stress and significant ultrastructural impairment of mitochondrial morphology with a loss of internal cristae, particularly after the exposure to stress stimuli such as high-NaCl concentration or LPS (10).

Furthermore, *NDUFC2* mRNA level was significantly downregulated, along with a higher degree of mitochondrial structural damage and dysfunction, in ACS compared to stable angina patients (7). *In-vitro, NDUFC2* silencing favored the endothelial expression of tumor necrosis factor α (TNFα), intercellular adhesion molecule 1 (ICAM), vascular cell adhesion molecule 1 (VCAM), matrix metallopeptidase 9 (MMP9) and CD40 ligand (CD40L) (7), all markers of inflammation, atherogenesis and plaque instability (23).

The results of the present study strongly suggest that the carrier status of the TT genotype at the *NDUFC2*/rs11237379 is an independent predictor of risk for ACS.

The clinical characteristics of our population were comparable with those of previously reported CAD patients' cohorts, with a higher prevalence of common risk factors for CAD, namely hypertension, hypercholesterolemia, and diabetes. The latter turned out to be a significant risk factor for recurrence of ACS in our study sample, whereas both hypertension and dyslipidemia were not (likely because of the ongoing medical treatments). Regarding the impact of therapy, the use of statins was confirmed as a protective strategy for secondary prevention of ACS (24).

Based on the previous evidence and the current results, the knowledge of the carrier status for the TT genotype at *NDUFC2*/rs11237379 may contribute to identify a more complete CV risk profile of affected patients and may provide a straightforward indication for more aggressive treatment strategies as well as for more frequent clinical monitoring, potentially improving the management of patients with CV risk factors and history of CV diseases.

The principal limitations of our study include the relatively small sample size, the single center design, the inclusion of only Caucasian patients and the lack of a control group represented by CAD patients without ACS.

## Conclusions

In a prospective cohort of Italian CAD patients, the TT *NDUFC2*/rs11237379 genotype appears to predispose to earlier ACS occurrence in carrier subjects. Remarkably, patients experiencing ACS have a significantly increased risk to develop new coronary events. In this regard, the carrier status for the TT genotype may represent a novel genetic CV risk factor. Overall, our findings support the role of *NDUFC2* deficiency, and of the consequent complex I-dependent mitochondrial dysfunction, as a relevant contributory mechanism to the development of endothelial and vascular damage and of atherothrombotic events ending with ACS.

Further studies are certainly needed to confirm and strengthen the current findings and to support a potential indication for screening the allele/genotype configuration at the *NDUFC2*/rs11237379 variant in CAD patients in the attempt to improve both clinical and therapeutic management.

## Data Availability Statement

The original contributions presented in the study are included in the article/supplementary materials, further inquiries can be directed to the corresponding author.

## Ethics Statement

The studies involving human participants were reviewed and approved by Ethical Committee of the S. Andrea Hospital. The patients/participants provided their written informed consent to participate in this study.

## Author Contributions

GG and SM selected the patients and performed the statistical analysis. GG wrote the manuscript draft. MC, RS, FB, and SM performed the genetic analysis. SB selected the patients and performed the follow-up. MV and CA revised the manuscript. SR conceived the work, revised the manuscript, and provided the funding. All authors contributed to the article and approved the submitted version.

## Funding

This work was supported by a grant from the Italian Ministry of Health, by a University Sapienza grant (project number RM1181641BF8C865), by Progetto PRIN 2017 (from the Italian Ministry of Instruction, University and Research, no. 2017PZY5K7).

## Conflict of Interest

The authors declare that the research was conducted in the absence of any commercial or financial relationships that could be construed as a potential conflict of interest.

## Publisher's Note

All claims expressed in this article are solely those of the authors and do not necessarily represent those of their affiliated organizations, or those of the publisher, the editors and the reviewers. Any product that may be evaluated in this article, or claim that may be made by its manufacturer, is not guaranteed or endorsed by the publisher.
